# Lucifensins, the Insect Defensins of Biomedical Importance: The Story behind Maggot Therapy

**DOI:** 10.3390/ph7030251

**Published:** 2014-02-27

**Authors:** Václav Čeřovský, Robert Bém

**Affiliations:** 1 Institute of Organic Chemistry and Biochemistry, Academy of Sciences of the Czech Republic, Flemingovo nám. 2, Prague 6, 16610 Czech Republic; 2 Diabetes Centre, Institute for Clinical and Experimental Medicine, Vídeňská 1958/9, Prague 4, 14021 Czech Republic; E-Mail: bemrob@yahoo.co.uk

**Keywords:** antimicrobial peptide, insect defensin, lucifensin, maggot therapy, *Lucilia sericata*, *Lucilia cuprina*, peptide isolation, peptide identification

## Abstract

Defensins are the most widespread antimicrobial peptides characterised in insects. These cyclic peptides, 4–6 kDa in size, are folded into α-helical/β-sheet mixed structures and have a common conserved motif of three intramolecular disulfide bridges with a Cys1-Cys4, Cys2-Cys5 and Cys3-Cys6 connectivity. They have the ability to kill especially Gram-positive bacteria and some fungi, but Gram-negative bacteria are more resistant against them. Among them are the medicinally important compounds lucifensin and lucifensin II, which have recently been identified in the medicinal larvae of the blowflies *Lucilia sericata* and *Lucilia cuprina*, respectively. These defensins contribute to wound healing during a procedure known as maggot debridement therapy (MDT) which is routinely used at hospitals worldwide. Here we discuss the decades-long story of the effort to isolate and characterise these two defensins from the bodies of medicinal larvae or from their secretions/excretions. Furthermore, our previous studies showed that the free-range larvae of *L. sericata* acutely eliminated most of the Gram-positive strains of bacteria and some Gram-negative strains in patients with infected diabetic foot ulcers, but MDT was ineffective during the healing of wounds infected with *Pseudomonas* sp. and *Acinetobacter* sp. The bactericidal role of lucifensins secreted into the infected wound by larvae during MDT and its ability to enhance host immunity by functioning as immunomodulator is also discussed.

## 1. Introduction

Over the course of their evolution, insects have developed an amazing resistance to bacterial infection, resulting in exceptional adaptation to a variety of natural environments often considered rather unsanitary by human standards. Insects respond to bacterial challenge or injury by rapid production of antimicrobial peptides (AMPs) that have a broad spectrum of activity against Gram-positive and Gram-negative bacteria and fungi. These peptides are evolutionary conserved components of the host’s innate immune system that form the first line of defence against infections and have been identified in almost all classes of life. Among the more than 2,000 AMPs listed in the Antimicrobial Peptide Database [1], peptides isolated from insects comprise the most abundant group. AMPs are synthesised in the fat body (the equivalent of the mammalian liver), epithelial cells, and in the certain cells of the haemolymph (the equivalent of mammalian blood) and then spread by the haemolymph over the entire body to fight infection [2]. The majority of these peptides belong to the class of cationic AMPs of molecular masses below 5 kDa [3]. Upon interacting with biological membrane or environments that mimic biological membranes, such as artificially made liposomes or sodium dodecyl sulfate, most are able to fold into highly amphipathic conformations with separated areas rich in positively charged and hydrophobic amino acid residues on the molecular surface [3,4,5]. The frequent occurrence of positively charged amino acid residues (Arg, Lys) in their molecules allows them to interact with the anionic phospholipids of bacterial membranes. This is followed by integration of the peptides into the lipid bilayer and disruption of the membrane structure via different modes that lead to leakage of cytoplasmic components and cell death [4,5,6]. Some studies have revealed that the killing process may proceed with relatively little membrane disruption but occurs rather by interfering with bacteria metabolism or interactions with putative key intracellular targets [7]. In contrast to conventional antibiotics, AMPs do not appear to induce microbial resistance and require only a short time to induce killing [6].

The AMPs isolated from insects may be classified on the basis of their sequence and structural features into three categories: (i) linear peptides which can form an α-helical structure and do not contain cysteine residues, such as cecropins; (ii) cyclic peptides containing disulfide bridges of which defensins are the most typical example and (iii) linear peptides with noticeable high content of one or two amino acid residues, mostly proline and/or glycine residues (pyrrhocoricins and diptericins) [2]. In this study, we will focus on the lucifensins [8,9]—two almost identical cyclic peptides of 40 amino acids residues and three intramolecular disulfide bridges belonging to the widely distributed family of insect defensins [10,11]. Lucifensin are the key antimicrobial peptides involved in the defence system of the blowfly larvae *Lucilia sericata* and *Lucilia cuprina*. These fly larvae are routinely used at hospitals worldwide during a procedure known as maggot debridement therapy (MDT) [12,13].

## 2. Insect Defensins

The first insect defensins were isolated from an embryonic cell line of *Sarcophaga peregrina* (flesh fly) [14] and from the haemolymph of immunised larvae of the black blowfly *Phormia terranovae* [15]. Since then, more than 70 defensins have been identified in various arthropods such as spiders, ticks, scorpions and in every insect species of the orders Diptera, Lepidoptera, Coleoptera, Hymenoptera, Hemiptera and Odonata investigated to date [10,11]. The defensins isolated from insects are 33 to 46 amino acid residues long with a few exceptions, such as the N-terminally extended defensin from the fly *Stomoxys calcitrans* [16] and C-terminally extended defensin found in the bee [17] and bumblebee [18]. They show sequence similarities ranging from 58 to 95% [2]. They may be further classified in two sub-families according to their *in vitro* activity against bacteria or filamentous fungi [11]: antimicrobial defensins that possesses activity against Gram-positive bacteria, including human pathogens, but are less effective against Gram-negative bacteria and fungi, and antifungal defensins that are mainly effective against filamentous fungi. Structurally, insect defensins possess an N-terminal flexible loop, a central α-helix and a C-terminal anti parallel β-sheet as has been determined by two-dimensional ^1^H-NMR spectroscopy carried out on isolated *Sarcophaga peregrina* defensin [19] and on a recombinant *Phormia terranovae* defensin [20]. The antimicrobial defensins contain six cysteine residues engaged in a characteristic conserved motif of three intramolecular disulfide bridges connected in a Cys1-Cys4, Cys2-Cys5 and Cys3-Cys6 pattern. On the other hand, the antifungal defensin drosomycin from *Drosophila* encompasses an additional short terminal β-strand and four disulfide bridges [21]. With the exception of royalisin, the defensin of the royal jelly of the honeybee [17] and bumblebee defensin [18], the C-terminal residue of insect defensins is not amidated. Although insect defensins were originally thought to be structurally similar to mammalian defensins, their three-dimensional structure and disulfide bridges pattern are different.

## 3. Maggot Therapy

Maggot debridement therapy is a controlled application of cultured sterile larvae of the flies *L. sericata* or *L. cuprina* to an infected chronic non-healing wound, especially in patients with impaired healing due to underlying disorders (e.g., diabetes and cardiovascular disease). The maggots gently and completely remove necrotic tissue by mechanical action (debridement) and by proteolytic digestion over 3–5 days of application. They rapidly eliminate infecting microorganisms which pass through their digestive tract [22], stimulate wound granulation and repair and thus enhance the healing process [12,13]. In addition, the larvae both secrete (by salivary glands) and excrete into the wound numerous substances including antimicrobial compounds, and alkalise the wound environment [23].

Since the introduction of maggot therapy into clinical practice by Baer [24], many researchers, influenced by successful therapeutic experience, have been focusing on the identification of antimicrobial agents secreted/excreted by maggots in the infected wound. It is quite surprising that up to now only a few active compounds have been identified in maggot excretions/secretions (ES) with explicitly determined chemical structures. These compounds include low molecular mass organic compounds and recently discovered insect defensins—lucifensins [8,9].

## 4. The Brief History of the Search for Antimicrobial Agents in Medicinal Larvae

Starting in the 1930s, researchers began to investigate the underlying mechanisms which may be responsible for some of the beneficial effects of maggot therapy. The main focus of interest has been examining the antimicrobial activity of the components of larval secretions and faecal waste products. In one of the initial studies of Simmons [25], published in 1935, it was found that the excretions obtained from the washings of the non-sterile *L. sericata* maggots exhibited considerable antimicrobial activity against several species of pyogenic bacteria which were killed during five- to ten-minutes of exposure. The activity of the excretion was not destroyed by autoclaving. In the research carried out two decades later by Pavillard and Wright [26], the washings of maggots combined with a suspension of their excretions were fractionated using paper chromatography. The active fraction was active against *S. aureus*. By means of a cellulose column and a modification of the chromatography technique, it was possible to obtain relatively pure samples of the antibiotic fraction. A series of injections of this preparation protected mice from the lethal effects of intraperitoneal inoculation with pneumococci. The final purification of this active compound was never implemented. Subsequent research done at several laboratories has demonstrated that larval excretions/secretions (ES) of *L. sericata* contain a variety of alkaline components inhibiting bacterial growth and that the pH increase provides optimal conditions for the activity of larvae-secreted proteolytic enzymes that liquidise necrotic tissues [23]. It also has been proposed that larvae release antimicrobial ingredients into the wound in response to infection. Some of these ingredients are bacteriostatic low molecular weight compounds such as *p*-hydroxybenzoic acid, *p*-hydroxyphenylacetic acid, proline dioxopiperazine [27] or an “enigmatic compound” of the empirical formula C_10_H_16_N_6_O_9_ known as the antibiotic seraticin [13]. The other compounds may possibly be antimicrobial peptides originating from the larval immune system which are released into the wound and thus contribute to wound healing [28,29]. These peptides belong to the groups of insect defensins, cecropins and diptericins [10,11].

Since 2000, several research groups have been aiming to isolate and characterise such antimicrobial peptides from the ES by utilising current methods of protein purification. In the laboratory of Bexfield [29], the ES of maggots was fractionated using an ultrafiltration device with a 10 kDa and 500 Da molecular weight cut-off membrane generating three fractions of molecular weights: >10 kDa, 500 Da–10 kDa and <500 Da. The activity against *S. aureus* was detected in <500 Da fraction and 500 Da–10 kDa fraction, but not in the fraction above 10 kDa. Even though these fractions were investigated in further detail regarding their physicochemical properties and antimicrobial activities [30], their constituents were not identified. The antimicrobial properties of *L. sericata* larval ES and the attempts to characterise its components were independently studied in several other laboratories [31,32]. For example, the study of Kerridge *et al.* [32] revealed in the secretions the presence of small (<1 kDa) antimicrobial factors active against Gram-positive bacteria such as *S. aureus*, including both methicillin-resistant *S. aureus* (MRSA) and methicillin-sensitive *S. aureus* (MSSA), and *Streptococcus pyogenes*. However, Gram-negative *Pseudomonas aeruginosa* was less sensitive. This active factor passed through the filter of the 3 kDa cut-off when the secretion was fractionated by ultrafiltration procedure. In this case, anti-MRSA activity was also detected in the retenates of the 10 kDa and 5 kDa filters indicating the presence of at least one additional larger antimicrobial agent. The authors concluded that the activities in the secretions possess characteristics consistent with insect antimicrobial peptides and are considered to be of low molecular weight, highly stable and a systemic part of the larva [32].

In 2013, Chinese researchers described the isolation of antimicrobial protein from an extract of the homogenate of *L. sericata* larvae using an ultrafiltration procedure [33]. The crude material obtained was named “antibacterial protein from maggots” (MAMP). MAMP demonstrated inhibitory activity against both standard strains and clinically isolated antibiotic-resistant strains of *S. aureus* *in vitro*. The topical use of MAMP effectively decreased the viability of *S. aureus* and promoted wound healing in an *S. aureus* mouse skin infection model. Although the authors claim the molecular weight of MAMP to be lower than 10 kDa, neither the chemical structure nor other specific identification of this “protein” was published.

Russian researchers [34] detected several inducible antimicrobial compounds by the “chromato-mass-spectrometry” method in the *L. sericata* larvae haemolymph and in the exosecretion released by the larvae. According to the authors, some of these compounds correspond to insect defensins and diptericins. Particularly, the molecular mass 4,117 Da of the peptide detected in the haemolymph matches well the molecular mass of lucifensin [8]. All the other compounds were identified solely based on their molecular masses, but their primary structures were not determined.

## 5. Lucifensin—The Defensin from *L. sericata*

### 5.1. Purification and Sequence Determination

Since 2007, we have been engaged in the identification of *L. sericata* AMPs by focusing on insect defensins. We have aimed to detect defensins in larval ES as well as different parts of the larval bodies, purify them and determine their primary structure. In our experience, it is evident that only the use of modern separation techniques such as high performance liquid chromatography (HPLC) as a part of the purification procedure may result in the discovery of the sought peptides.

The physicochemical properties of insect defensins (medium size, cationic molecule, contains disulfide bridges) influenced us in the selection of the purification procedure. Starting with the extractions of *L. sericata* larval guts, a strongly acidic acetonitrile/water/0.5% trifluoroacetic acid mixture, which provides good solubility for cationic peptides while protecting its stability against enzymatic digestion and disulfide bridges reshuffling, was the extraction solvent of choice. Successive ultrafiltration of crude extract, the size exclusion HPLC and following reversed phase HPLC (RP-HPLC) applied as the final steps of the purification procedure resulted in the peptide of the purity satisfactory for sequencing by Edman degradation [8].

The Edman degradation using 40 cycles yielded the following N-terminal sequence: Ala-Thr-X-Asp-Leu-Leu-Ser-Gly-Thr-Gly-Val-Lys-His-Ser-Ala-X-Ala-Ala-His-X-Leu-Leu-Arg-Gly-Asn-Arg-Gly-Gly-Tyr-X-Asn-Gly-Arg-Ala-Ile-X-Val-X-Arg-Asn, assuming that all six undetermined amino acid residues (X) were cysteines. The molecular mass of this defensin measured by ESI-QTOF MS was determined to be 4,113.6. This was in good agreement with the calculated value of 4113.89, based on the sequence determined by Edman degradation and assuming that the six cysteine residues form three disulfide bridges [8]. Our results showed that *L. sericata* defensin, which we term lucifensin, differs from *Phormia terranovae* defensins A and B and from *Sarcophaga peregrina* sapecin by five amino acid residues (Val11, Lys12, Arg33, Ala34, and ILe35).

Knowing the properties of lucifensin, we were able to detect its presence in the extracts of other larval tissues such as the salivary glands, fat body, haemolymph as well as in the larval ES [8]. However, no antimicrobial peptide from other families such as cecropins, diptericins or Pro-rich peptides was detected in the frame of our study.

### 5.2. Synthetic Lucifensin

In 2011, we reported a total chemical synthesis of lucifensin using the methodology of solid phase peptide synthesis [35]. In the first step of the synthesis, we prepared the linear peptide of 40 amino acid residues containing six cysteines in the sequence. Oxidative folding of this linear peptide yielded a cyclic peptide with the disulfide bridges formed between Cys3-Cys30, Cys16-Cys36 and Cys20-Cys38; this disulfide bridges pattern corresponds to that of natural lucifensin.

Synthetic lucifensin was highly active against *M. luteus* and *Bacillus subtilis* with MIC values of 0.6 and 1.2 µM, respectively, while lower but significant activity was observed against *S. aureus* with MIC value of 41 µM. No activity was detected against *E. coli*, thus confirming the generally recognised fact that insect defensins are more active against Gram-positive than Gram-negative bacteria. The peptide showed slight antifungal activity against *C. albicans* (MIC = 86 µM) and was not haemolytic against human red blood cells [35]. These findings corresponded to the clinical effect of maggot therapy and supported our hypothesis that lucifensin is the long-sought antimicrobial factor of medicinal maggots.

To confirm the importance of disulfide bridges for its activity and structure, we synthesised three lucifensin analogs, each of which was cyclised through only one native disulfide bridge in different positions and having the remaining four cysteines substituted by alanine [35]. The analog cyclised through a Cys16-Cys36 disulfide bridge showed weak antimicrobial activity, while the other two analogs containing one disulfide bridge were inactive. These results indicate that the presence of disulfide bridges in lucifensin is essential for its antimicrobial activity as it is necessary for preserving its three-dimensional structure. The synthesis of truncated lucifensin at the N-terminal by 10 amino acid residues resulted in an almost inactive analog [35].

### 5.3. Three Dimensional Structure and Mode of Action

**Figure 1:**
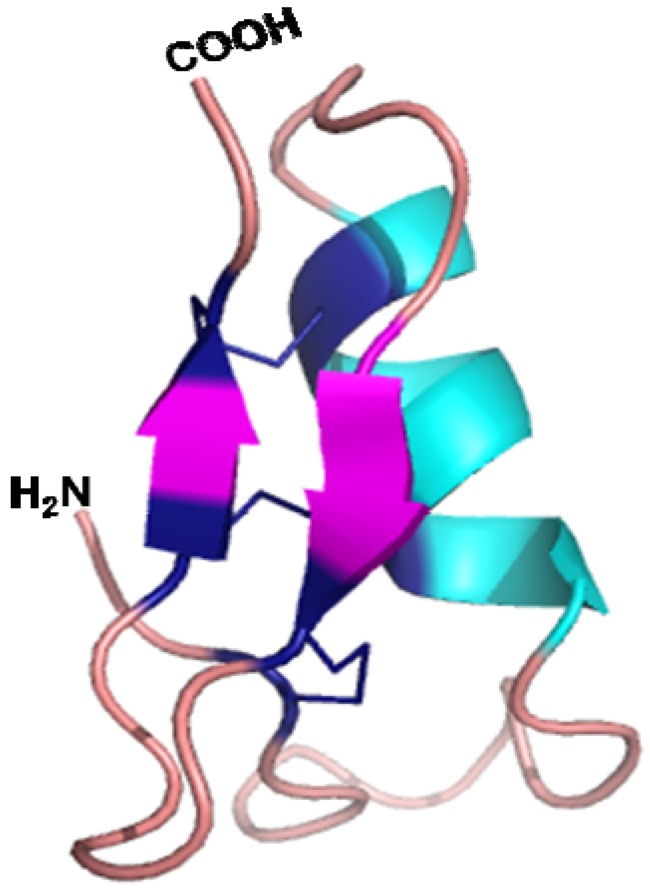
An illustrated representation of the three-dimensional structure of lucifensin (*L. sericata* defensin) which was generated in Pymol [38] by using the solution structure of lucifensin (PDB code 2LLD).

The tertiary structure of lucifensin determined using NMR [36] showed a high degree of similarity to the structure of other insect defensins: sapecin [19] and insect defensin A [20]. Lucifensin adopts a characteristic insect defensin structure that includes an N-terminal loop (residues 1–12), followed by an α-helix (residues 13–23), which is linked by a turn to a pair of β-strands (residues 28–31 and 34–38) folded into an antiparallel β-sheet (Figure 1). The Cys3-Cys30 disulfide bridge connects the N-terminal loop with the first β-strand and the other two bridges (Cys16-Cys36, Cys20-Cys38) link the α-helix and second β-strand [36]. The α-helix and β-structure connected by two disulfide bridges form a common structural element typical for insect defensins, known as the cysteine-stabilised αβ (CS αβ) motif, which is essential for their antimicrobial activity [19,20].

The action mechanism of lucifensin relates to the study on homologous sapecin—the defensin of *Sarcophaga peregrina* for which a putative mechanistic model for membrane permeabilisation has been already proposed [37]. According to this model obtained on the basis of NMR experiments, sapecin oligomerises in the bacterial membrane and thus forms the channels therein which results in consequent leakage of cytoplasmic components and bacterial cell death. This putative model of sapecin oligomerisation is based on an electrostatic interaction between Asp4 of one sapecin molecule and Arg23 of another sapecin molecule, as these two residues are situated at opposite ends of the oligomerisation site. Since the sequences of the lucifensin differs from that of sapecin by only four amino acid residues (positions 11, 12, 33 and 35) and residues Asp4 and Arg23 are conserved in lucifensin, we may speculate that the mechanism of the lucifensins antimicrobial action is the same as that proposed for sapecin [37]. We may then suppose that the absence of Asp4 in the truncated analog of lucifensin might be the reason that its antimicrobial activity significantly decreased. 

**Figure 2:**
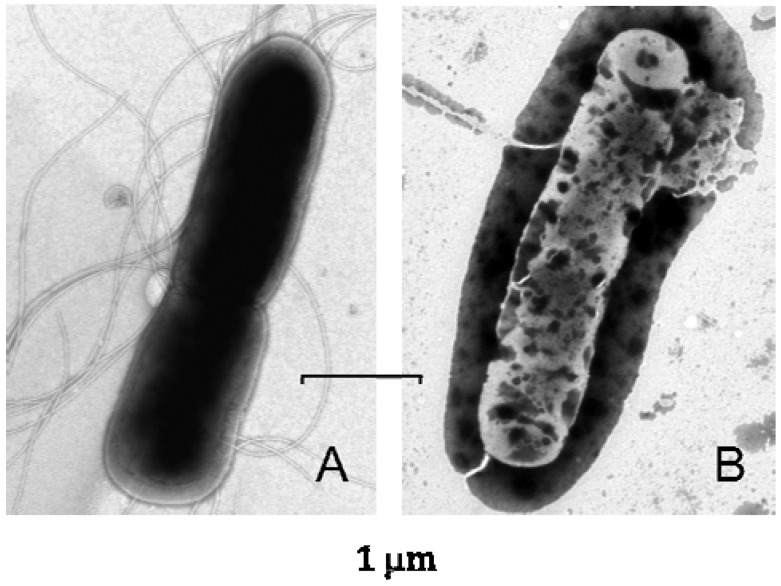
Electron micrographs of negatively stained *Bacillus subtilis* either untreated (**A**) or treated by lucifensin for 60 min (**B**). Scale bars represent 1 µm.

As illustrated in Figure 2, the treatment of *B. subtilis* by lucifensin followed by transmission electron microscopy revealed significant changes in the bacterial envelope leading to final breakup of bacterial cells [35], just demonstrating the generally accepted mechanism of the action for cationic antimicrobial peptides, such as insect defensins.

## 6. Lucifensin II—The Defensin from *L. cuprina*

The homolog of insect defensin designated lucifensin II was recently isolated from an extract of the haemolymph of the fly larva *Lucilia cuprina* in our laboratory [9]. We applied an improved purification procedure comprising of two ultrafiltration steps, RP-HPLC, modified size exclusion HPLC and a final RP-HPLC purification leading to the successful determination of its full-length primary sequence. This sequence determined by ESI-orbitrap mass spectrometry and Edman degradation shows almost the same identity to the sequence of lucifensin (*Lucilia sericata* defensin). The lucifensin II sequence differs from that of lucifensin by only one amino acid residue; that is by isoleucine instead of valine at position 11. The presence of lucifensin II was also detected in the extracts of other larval tissues such as gut, salivary glands, fat body and whole body extract [9].

We isolated lucifensin II from the haemolymph of non-sterile maggots which has led to the question of whether lucifensin II is produced in response to poly-microbial challenge or is it constitutively expressed by the larval immune system. Accordingly, we analysed the anti-*M. luteus* active RP-HPLC fractions of 50 kDa filtrate obtained either from the haemolymph of non-sterile maggots or haemolymph of maggots treated individually with the *S. aureus*, *P. aeruginosa* and *Proteus mirabilis*, by mass spectrometry. The presence of lucifensin II was detected in all corresponding fractions independently of whether the maggots were challenged by infection or were kept sterile [9]. This observation is not in agreement with a hypothesis predicting no antibacterial activity in larvae without bacterial challenge [39]. In addition, we were not able to detect any other cationic antimicrobial peptides in the haemolymph of *L. cuprina* in the course of lucifensin II purification.

## 7. Molecular Biology Approaches for the Identification of Lucifensin in Medicinal Larvae

Using suppression subtractive hybridisation methodology, Altincicek *et al.* [40] identified numerous genes that are up-regulated in larvae of *L. sericata* upon septic injury. These genes encode signalling proteins, proteinases and homeostasis proteins and also potential antimicrobial peptides. The deduced peptides share sequence similarities with insect defensins, diptericins and proline-rich peptides which are conserved within Diptera. However, none of these deduced sequences match to that of lucifensin.

Danish researchers used for the identification of lucifensin in *L. sericata* maggots a transposon-assisted signal trapping, a methodology specially developed for identification of secreted proteins and peptides. They applied this method to *L. sericata* maggots induced with external stimuli mimicking those encountered by the maggots during MDT [41]. The lucifensin sequence determined in that laboratory [41] was identical to that published by us [8]. They also produced a few milligrams of recombinant peptide and estimated its antimicrobial activity against both Gram-positive and Gram-negative bacteria. Lucifensin was active against *S. carnosus*, *Streptococcus pyogenes* and *Streptococcus pneumoniae* with MIC values of 2 mg/L, and against *Enterococcus faecalis* and *S. aureus* with MIC values of 32 and 16 mg/L, respectively, but did not show any antimicrobial activity towards the Gram-negative bacteria tested at concentrations **<**128 mg/L. The MIC of lucifensin for a selection of 15 MRSA and glycopeptide-intermediate *S. aureus* isolates tested ranged from 8 to 128 mg/L [41].

The expression of lucifensin in various larval tissues during *L. sericata* development and in maggots exposed to infections was recently examined by Slovak researchers [42]. Using an *in situ* hybridisation method, they revealed lucifensin expression in the salivary glands of all larval stages. No differences were detected in the salivary glands after stimulation by bacteria. However, lucifensin expression was strongly stimulated in the fat body in response to the infectious environment and it was found that it is secreted solely from this tissue into the haemolymph [42].

## 8. Lucifensin Released by Maggots to the Wound

**Figure 3:**
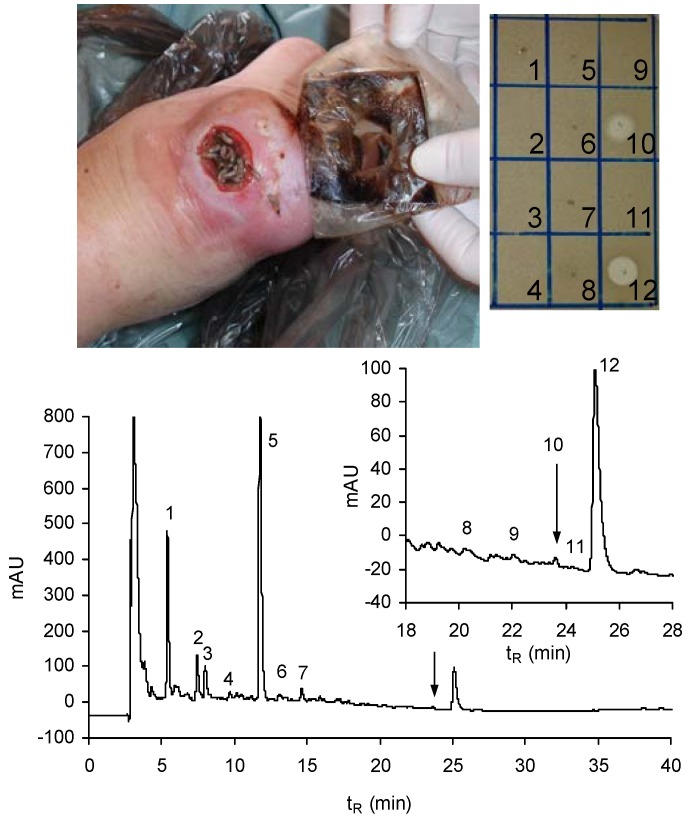
RP-HPLC profile (at 220 nm) of the lyophilised filtrate obtained by ultrafiltration through 10 kDa cut-off membrane of the swab extract taken from the wound (photo) immediately after removal of the larvae. An elution gradient of solvents from 5% to 70% acetonitrile/water/0.1% TFA was applied for 60 min at a flow rate 1 mL/min. Arrows indicate the anti-*M. luteus* active peak (10) containing lucifensin. The larger peak at t_R_ = 25 min (12) represents a mixture of two human α-defensins: HNP1 and HNP2. Inset: Anti-*M. luteus* activity (clear zones in the drop diffusion test) of selected peaks delineated in the profile.

We analysed the extract of the swabs taken from the infected diabetic foot ulcers (DFU) during maggot treatment or immediately after removal of the maggots from the wound (Figure 3). The extracts of these samples were pre-purified by ultrafiltration through 10 kDa molecular weight cut-off membrane to remove high molecular mass components and then the filtrates were lyophilised. The HPLC profile of obtained material (Figure 3) indicates the presence of a tiny amount of lucifensin together with two human α-defensins (HNP1 and HNP2). In a drop diffusion test against *M. luteus*, the fraction of lucifensin exhibited almost equal antimicrobial activity against Gram-positive bacteria as the fraction corresponding to the mixtures of these two HNPs (Figure 3). These two host defence peptides were apparently produced and released into the wound by the components of the human immune system, including some blood cells (neutrophils) as the innate immune response against infection.

The antimicrobial effect of maggots was investigated in several *in vivo* studies by comparing bacterial diversity in the wounds before and after their application. Our study in 91 patients with DFU demonstrated that maggot therapy by free-range larvae applied to the wound for an average 3 days acutely eliminated most of the Gram-positive and Gram-negative strains including methicillin-resistant *Staphylococcus aureus* (MRSA), but maggots were ineffective against *Pseudomonas* sp. and *Acinetobacter* sp. [43]. The antimicrobial effect persisted 7–13 days after removal of the larvae. These results are in accordance with the observations of other researchers [31,44] and signify that lucifensin, as an external antimicrobial peptide presented in DFU, may play a key role as a microbicide and as a healing factor in the majority of maggot-treated DFU. In cases of ulcers infected by *P. aeruginosa* or some other Gram-negative bacteria, we hypothesize that maggot therapy fails due to the specific activity of lucifensin against Gram-positive bacteria.

In addition to killing bacteria directly, lucifensin and other antimicrobial compounds from maggots have a number of immunomodulatory functions that may be involved in the clearance of infection and support of wound healing, including the ability to influence host innate and adaptive immune response. Regarding the innate immune system, a wide variety of human antimicrobial peptides is expressed by the epidermal cells and neutrophils, such as human β-defensins and HNPs [45]. Besides their antimicrobial effect, AMPs also support processes of wound healing, such as proliferation and angiogenesis or keratinocyte migration [46]. In contrast to acute wound healing, chronic wounds are marked by a prolonged and dysregulated inflammatory phase. Inflammatory cells like neutrophils, monocytes and macrophages are not only present in excess numbers, they also have enhanced production and release of pro-inflammatory cytokines, proteases and reactive oxygen species, leading to growth factor inactivation and tissue destruction [47]. Moreover, chronic ulcers from diabetic patients showed β-defensins up-regulation; the production of these antimicrobial peptides might be insufficient to mount proper antimicrobial control and wound healing [48]. Lucifensin and other antimicrobial compounds from maggots may turn this unfavourable situation and transfer the wound to satisfactory healing. Maggot secretions potently inhibit the pro-inflammatory responses of human neutrophils without affecting their antimicrobial activities [49]. In addition to reducing the production of proinflammatory cytokines and host antimicrobial peptides, maggot secretions also increased the production of pro-angiogenic growth factors bFGF and VEGF in anti-inflammatory macrophages [50]. Simultaneously, the increased pro-angiogenic activity of anti-inflammatory macrophages may induce neovascularisation and the concurrent formation of granulation tissue. In addition, maggots increase the expression of bFGF in ulcer tissue and induce the formation of granulation tissue.

## 9. Perspectives on the Future of Lucifensins

Bacterial resistance to conventional antibiotics is a major concern and the main reason for extensive, ongoing research to develop new therapeutics. Antimicrobial peptides could both affect the pathogens and simultaneously activate and modulate innate and adaptive immune systems of the host. Lucifensins have interesting features for topical application to treat wound infection and promote wound healing. These peptides that act simultaneously on the pathogens as well as on the host offer a unique opportunity to minimise the direct selective pressures for pathogen resistance. For lucifensin, there are several different potential strategies for its therapeutic application: (i) as single anti-infective agent, (ii) in combination with conventional antibiotics, (iii) in combination with other antimicrobial peptides. The reason for using lucifensin with conventional antibiotics is a formation of bacterial biofilm in the wound. Bacteria within chronic wounds often reside in biofilms that protect them from antibiotics and the immune system. A combination of lucifensin and antibiotics may ensure complete breakdown of the biofilms, thereby preventing bacterial re-growth from the remaining matrix, and prompt antibiotic action against the bacteria released from the biofilms. Preclinical studies with lucifensin for testing of safety, pharmacokinetics and toxicity are needed. After that, clinical studies may be initiated.

## 10. Conclusions

We propose that lucifensins are key antimicrobial factors involved in the defence system of medicinal larvae *L. sericata* and *L. cuprina* which protect maggots when they are exposed to the highly infectious environment of a wound during maggot therapy. They act as a microbicide and healing factor within the wound. Their discovery as a crucial disinfectant secreted/excreted by maggots to the wound broadened the understanding of the healing mechanism of maggot therapy. As the deliberate treatment of non-healing wounds by maggots has been in practice since the 1930s, can we possibly consider lucifensin as a prime example of the practical application of antimicrobial peptide in medicine?
